# Tubular Ectasia of the Epididymis: A Case Report and Literature Review

**DOI:** 10.7759/cureus.68169

**Published:** 2024-08-30

**Authors:** Aditya S Pedaprolu, Mehak F Semy, Abhilasha Bhargava

**Affiliations:** 1 General Surgery, Datta Meghe Institute of Higher Education and Research, Wardha, IND; 2 Internal Medicine, Dr. D. Y. Patil University, School of Medicine, Mumbai, IND

**Keywords:** urology, benign epididymal cyst, tubular ectasia, epididymis, ectasia

## Abstract

Tubular ectasia of the epididymis is a rare benign disorder that, when present, is usually seen in patients post-vasectomy. It can also be seen in patients with a prior history of trauma or local infection. Patients typically present with a palpable scrotal mass and dull-aching pain. It is important to differentiate this disease from other common disorders such as varicocele, hydrocele, spermatocele, or even testicular malignancy. We report a rare case of tubular ectasia of the epididymis in a 55-year-old patient with palpable scrotal swelling with no prior operative history of vasectomy nor any history of trauma. We share this patient's ultrasonography findings along with a literature review of this uncommon disease.

## Introduction

The testes are the male reproductive and endocrine organs responsible for spermatogenesis and production of testosterone. The spermatozoa produced by the testis exit through a series of ductal structures that emerge into the rete testis, efferent ductules, epididymis, and vas deferens. The epididymis, located posteriorly and slightly lateral to the testis, serves as an area for the maturation of sperm before reaching the vas deferens and the ejaculatory duct [1]. An obstruction in the vas deferens due to various causes may lead to slow, progressive accumulation of sperms, leading to an increase in tenuous tubular pressure of the epididymis, thereby causing dilatation and cystic changes of the epididymis, which is referred to as tubular ectasia. Only 5% of intratesticular tumors, such as tubular ectasia, are benign, so they hold significant importance in clinical practice, particularly because one of the primary differential diagnoses is often a testicular neoplasm. This rare disorder is identified using ultrasonography and color Doppler imaging [2]. We report a case of tubular ectasia of epididymis and analyze their clinical presentation and investigations leading to diagnosis and subsequent management.

## Case presentation

A 55-year-old male came to our outpatient department with complaints of left-sided hemi-scrotal swelling, occasionally accompanied by pain for the past three months. The pain was of a vague "dragging" type (rated 1 to 2 on the numerical pain scale), which was non-radiating and subsided on its own. There was no history of altering the size of the swelling or aggravation of pain while standing, lying down, walking, or engaging in strenuous activities. The patient had no history of trauma, subfertility, malaise, weight loss, or chronic infections such as filariasis or tuberculosis. He also reported no urinary complaints, such as a "burning" sensation during micturition, an increase in frequency, nocturia, urgency, or a poor stream during micturition. Additionally, he had no previous surgical interventions, such as vasectomy, in the past. He had no known comorbidities. On clinical examination, we could see a 4 x 4 cm swelling, which was confined to the scrotum. No expansile impulse was noted on a cough. The skin over the swelling was normal, with no skin changes or signs of inflammation. On palpation, the swelling was soft, cystic, and non-tender. The spermatic cord was also felt, which was non-tender and normal. Left-sided testis was also separately felt, which was normal. The contralateral testis and genitalia were also normal. There was no evidence of any mass or lymph nodes in the abdomen or supraclavicular region.

**Figure 1:**
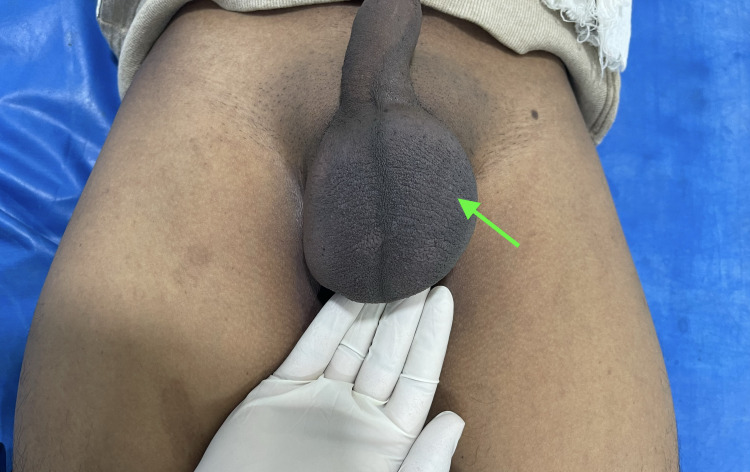
Clinical image of the patient with a left-sided swelling over the scrotum (green arrow).

**Figure 2:**
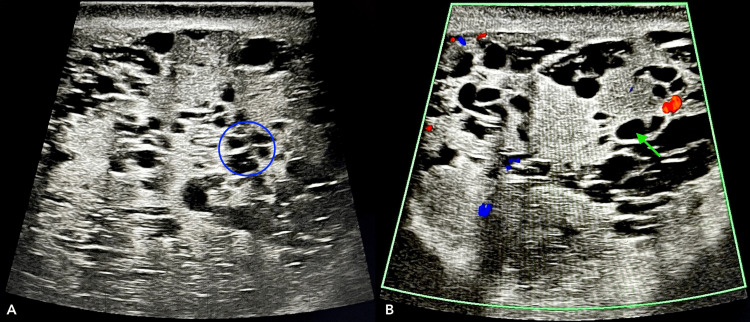
(A) Inguinoscrotal ultrasonography showing multiple cystic tubular structures seen in the testicular mediastinum (blue circle); (B) color Doppler showing these cystic structures (green arrow) taking vascularity on Doppler.

A routine urine microscopy was done, which was normal, showing no signs of infection or other issues. We then recommended that the patient undergo an inguinoscrotal ultrasound with color Doppler. The report indicated that the left epididymis was enlarged (4.5 x 4.5 cm), with multiple cystic tubular structures that lacked vascularity on Doppler (Figures 1, 2). Bilateral testes were normal in size, shape, and echotexture and demonstrated normal vascularity on Doppler. The final impression was tubular ectasia of the left epididymis.

Due to the condition's small size and relatively asymptomatic nature, we have chosen a conservative, "wait and watch" approach. The patient was started on an intravenous followed by an oral prophylactic course of β-lactam antibiotics and analgesics given as needed (SOS). After three days, he was discharged with instructions to follow up in one month.

## Discussion

Tubular ectasia typically occurs in those with a prior operative history of vasectomy or excision of an epididymal cyst due to chronic accumulation of sperms in the tubules of the epididymis. However, it can also be seen with other causes of obstruction of the vas deferens, such as infection (urinary tract infections or sexually transmitted diseases) or trauma [3]. Cysts and cystic dilatation of epididymis and rete testis constitute the most common paratesticular cystic lesions, frequently in men > 55 years old and seen in up to 30% of asymptomatic patients at ultrasound [4]. This disorder can be identified using ultrasonography, where a characteristic speckled appearance is noted due to multiple fluid-filled interfaces between the tubules of the epididymis [5]. Another feature that can be seen is "dancing megasperm," where tiny echogenic foci are seen continuously oscillating within the dilated tubules of the epididymis [6]. Cystic dysplasia of the rete testis is rare, generally unilateral, most often seen in children (mean presentation of 5 years old), and associated with urinary tract malformations. In contrast, tubular ectasia of the epididymis is seen commonly in patients in the sixth decade of life [7].

Some common benign differentials of tubular ectasia are varicocele, epididymal cyst, or spermatocele. A varicocele can be differentiated by clinical examination (classical presentation of "bag of worms" either on normal palpation or on cough impulse) or color Doppler imaging, which shows no flow in case of tubular ectasia [8]. Epididymal cysts are often bilateral, usually posterior to, but separate from the testis. Spermatoceles are unilocular retention cysts located in the epididymal head above and behind the upper pole of the testis. Diagnosis for either of these cysts can be easily made using ultrasonography.

Another significant feature of this disorder is that it must be differentiated from a testicular malignancy as the clinical presentation is very similar. However, in ultrasonography, the malignant neoplasms may appear as solid lesions, while benign tubular ectasia appears as cystic. Further confirmation by magnetic resonance imaging (MRI) helps diagnose doubtful cases [9].

Management of tubular ectasia usually involves symptomatic treatment using broad-spectrum antibiotics and analgesics with surgical excision of an associated epididymal cyst or spermatocele or even epididymectomy is to be considered as a last resort if the problem continues to persist [10].

## Conclusions

Tubular ectasia of the epididymis is a benign lesion caused by obstruction at the tubules of the epididymis due to post-vasectomy, post-traumatic, or post-infectious causes. Diagnosis can be made using a thorough clinical history and examination, supplemented with radiological investigations such as ultrasonography with color Doppler to note the vascularity of these cystic lesions and differentiate them from other neoplastic or non-neoplastic lesions. Accurate diagnosis of this rare disease is crucial, as patients often present with minimal symptoms, allowing us to avoid unnecessary surgical interventions and manage this disease conservatively.
